# Lipid synthesis and secretion in HepG2 cells is not affected by ACTH

**DOI:** 10.1186/1476-511X-9-48

**Published:** 2010-05-17

**Authors:** Maria Skoog, Maria Berggren-Söderlund, Peter Nilsson-Ehle, Ning Xu

**Affiliations:** 1 Section of Clinical Chemistry & Pharmacology, Institute of Laboratory Medicine, Lunds University, S-221 85 Lund, Sweden

## Abstract

Apolipoprotein B (apoB) containing lipoproteins, i.e. VLDL, LDL and Lp(a), are consequently lowered by ACTH treatment in humans. This is also seen as reduced plasma apoB by 20-30% and total cholesterol by 30-40%, mostly accounted for by a decrease in LDL-cholesterol. Studies in hepatic cell line (HepG2) cells showed that apoB mRNA expression is reduced in response to ACTH incubation and is followed by a reduced apoB secretion, which may hypothesize that ACTH lowering apoB containing lipoproteins in humans may be mediated by the inhibition of hepatic apoB synthesis. This was recently confirmed *in vivo *in a human postprandial study, where ACTH reduced transient apoB48 elevation from the small intestine, however, the exogenic lipid turnover seemed unimpaired. In the present study we investigated if lipid synthesis and/or secretion in HepG2 cells were also affected by pharmacological levels of ACTH to accompany the reduced apoB output. HepG2 cells were incubated with radiolabelled precursors ([^14^C]acetate and [^3^H]glycerol) either before or during ACTH stimuli. Cellular and secreted lipids were extracted with chloroform:methanol and separated by the thin layer chromatography (TLC), and [^14^C]labelled cholesterol and cholesteryl ester and [^3^H]labelled triglycerides and phospholipids were quantitated by the liquid scintillation counting. It demonstrated that ACTH administration did not result in any significant change in neither synthesis nor secretion of the studied lipids, this regardless of presence or absence of oleic acid, which is known to stabilize apoB and enhance apoB production. The present study suggests that ACTH lowers plasma lipids in humans mainly mediated by the inhibition of apoB synthesis and did not via the reduced lipid synthesis.

## Introduction

ACTH (adrenocorticotrophic hormone) is primary involved in the regulation of steroid hormone synthesis in the adrenal gland [1,2]. When ACTH is administered exogenous it has proven to have positive function on neurological diseases such as infantile spasms and multiple sclerosis [3,4]. Over the last decade we have demonstrated that ACTH also has beneficial effects on lipid profile that is not accounted for by the induction of steroid hormone synthesis. ACTH systematically lowers all lipoproteins containing apolipoprotein (apo) B, i.e. very low-density lipoprotein (VLDL), low-density lipoprotein (LDL) and Lipoprotein (a), while high-density lipoprotein (HDL) rather is elevated [5]. Further a pronounced and consistent decrease of plasma total cholesterol in particular LDL-cholesterol by 30-40% is seen after ACTH treatment in healthy individuals and in patients with secondary hyperlipidemia [5-9]. Plasma phospholipids are also significantly reduced and triglycerides are mainly lowered, especially in patients with initially high levels [5,6,8-10].

Studies in vitro, in the HepG2 cell line, indicated that output of apoB and not elimination of apoB containing lipoproteins, via the liver, was the effect of ACTH. Thus, at 10 times physiological level, ACTH significantly and selectively suppresses apoB mRNA levels and apoB secretion, while the receptor mediated uptake of apoB containing lipoproteins, via the LDL receptor or scavenger receptor-BI, or the mRNAs of these receptors were unchanged [11]. The hypothesis that ACTH reduced apoB output was further supported in vivo, by using the human postprandial phase to study the induced apoB48 production. In healthy humans ACTH significantly inhibited the transient rise in apoB48 in plasma, while the lipid handling, followed as triglycerides and retinyl palmitate, seemed unimpaired [10]. We hypothesised that ACTH induced the intestinal cells to produce larger, more lipid rich, but fewer lipoproteins. To see if this is the case also in hepatic cells we herein studied the effects of ACTH on lipid handling i.e. synthesis and secretion in liver cell cultures where we know apoB output is reduced in response to ACTH.

## Materials and methods

### Materials

RPMI 1640 with L-glutamine, fetal calf serum (FCS), penicillin/streptomycin (10000 units/mL/10000 μg/mL) and trypsin-EDTA were obtained from Life Technologies. The hepatoblastoma cell line HepG2 was obtained from the American Type Culture Collection, Manassas, VA. Vented cell culture flasks, 25-cm^2 ^were from Nunc, Denmark. ACTH1-39 (porcine pituitary), oleic acid (OA), human serum albumin (HSA) and bovine serum albumin (BSA) were from Sigma. ACTH1-24 (Synacthen) was from Novartis and Insulin (Actrapid) was from Novo Nordisk. [1-^14^C]acetate (acetic acid-sodium salt, specific activity 57,0 mCi/mmol) and [2-^3^H]glycerol (specific activity 1 Ci/mmol) was from Amersham Pharmacia Biotech and scintillation liquid (OptiFluor) was from Perkin Elmer. Thin layer chromatograph plates, aluminium sheets with silica gel 60, were from Merck. Internal standards cholesterol, cholesteryl oleate, phosphatidylcholine and glyceryl trioleate was from Sigma. All other chemicals and solvents used were of analytical grade.

### Cell culture conditions

Stock cultures of HepG2 were kept in 25-cm^2 ^flasks under basal conditions. RPMI 1640 complemented with 100 units/mL penicillin and 100 μg/mL streptomycin and standard culture conditions (5%CO_2_, 37°C), with 10% FCS present. Medium was changed every second to third day. Cells were subcultured every seventh day, just prior to reaching confluence, into new stock culture and to experimental cultures. Cells were only used up to 20-25 subcultures and were regular controlled for mycoplasma presence. Into each experimental flask (25 cm^2^) 3 × 10^5^cells/mL were seeded. Medium was changed every second to third day and between incubations with different experimental media cells were washed twice with phosphate-buffered saline (PBS). During all the experiments cells were subconfluent and all experimental media contained penicillin/streptomycin and FCS was substituted with 0,5% HSA. Basal experimental media contained 0,4 mM oleic acid if not mentioned otherwise. Oleic acid is suggested to increase stability of apoB and hence increase synthesis of apoB containing lipoproteins [12]. Briefly, oleic acid (dried under nitrogen) and HSA (water solution), molar ratio 20:1, was strongly vortexed and was inversed over night at 8°C. The suspension was added to experimental media, containing 0,5% HSA, to obtain the final ratio 4:1 of oleic acid and HSA.

### Assay of lipid synthesis and secretion

Prelabel studies; HepG2 cells were incubated in experimental medium with radionuclides, [^14^C]acetate (0,5 μCi/ml) or [^3^H]glycerol (1 μCi/ml) for 20 hrs. Medium was replaced with experimental medium containing ACTH (10, 100 or 200 pM Synacthen1-24) or insulin (1 mg/ml (170 mM) Actrapid). Continuous labelling; HepG2 cells were preconditioned in the experimental medium with ACTH (100 pM ACTH1-39) for 24 hrs. Medium was replaced with experimental medium containing ACTH and [^14^C]Acetate (0,5 μCi/ml) or [^3^H]Glycerol (1 μCi/ml). During time media samples were collected and stored at -20°C. After 24 hrs cells were scraped into 1 mL of PBS.

### Extraction and analysis of lipids

Lipids were extracted from cell and media samples with [chloroform: methanol (1:2; v/v)] [13]. Briefly, step 1: Add [chloroform: methanol (1:2)] to obtain ratio 1:2:1 of [chloroform: methanol: water (sample and KH_2_PO_4_, 0,1 M, pH 7,45)]. Remove upper phase of water-soluble products. Step 2: Add chloroform and KH_2_PO_4 _to obtain ratio 2:1:1 of [chloroform: methanol: water]. Remove upper phase and wash lipid phase with equal volume of [chloroform: methanol: KH_2_PO_4 _(30:480:470)]. The lipid phase was dried under nitrogen gas, dissolved in a small volume of chloroform together with internal standards of unlabeled lipids (cholesterol, cholesteryl oleate, phosphatidylcholine and glyceryl trioleate). The samples were applied onto the TLC plates that were preactivated at 80°C over night. Lipids were separated in [petroleumether: diethyl ether: acetic acid (75:25:1; v/v)] for 45 min [14], and were visualised by iodine vapour. The spots were solved in 1 mL of methanol: water (1:1; v/v) and counted in 10 mL of scintillation liquid (OptiFluor) in a beta counter (Rack beta 1214).

### Recovery of [^14^C] radioactivity with prelabel design

After 20 hrs incubation with radioisotope the intracellular activity comprised 40% of the added activity. Of this cellular activity 83% is organically solved and after a 24 hrs chase period 81% is recovered from cells and media as cholesterol, cholesteryl ester, phospholipids, triglycerides and free fatty acids as separated by the TLC.

### Analysis of total protein

Total protein contents of cells were measured according to Lowry [15] with BSA as standard. Cells in PBS were lysed with addition of equal volume of NaOH (0,1 M). Protein content of cells did not differ between treated and untreated cells.

### Statistical analysis

Results are expressed as mean ± SD. Data were statistical analysed with GraphPad Prism software. ANOVA was used for comparisons of three groups with Bonferroni post-test for selected pairs of columns. Significant difference was established at p < 0.05.

## Results

**Figure 1:**
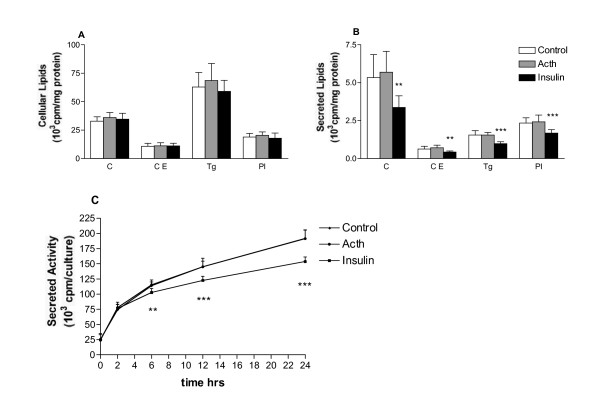
**Contents of labelled lipids in cells and culture media**. HepG2 cells prelabelled with [^14^C]acetate or [^3^H]glycerol was incubated with ACTH (200 pM Synacthen) and insulin (1 mg/ml actrapid). Labelled cholesterol, cholesteryl ester, triglyceride and phospholipid, respectively, were evaluated after 24 hrs in cells (A) and media (B). Total secretion of [^14^C] products was followed during the experiment (C). Data represent mean ± SD of two repeated experiments (N = 11). ** p < 0,01, *** p < 0,001.

Cells prelabelled with radionuclides were stimulated with ACTH (10, 100 or 200 pM) for 24 hrs and lipids were assessed in media and harvested cells. In the cells the lipid pools of de novo synthesised cholesterol, cholesteryl ester, triglycerides and phospholipids, from the radionuclide precursors, were not affected by the ACTH (Fig. 1A), and there was no difference between the groups pretreated by different ACTH concentrations (data not shown). Secretion of synthesised radiolabelled lipids at 24 hrs was also unaffected by the ACTH (Fig. 1B). Cells incubated with insulin as a positive control [16] showed a strong significant reduction of secreted lipids at 24 hrs, although the cellular pool was not changed. Over the 24-hour chase period, after prelabelling, the total output of labelled products into media was increasing, measured as total [^14^C] decays in the media (Fig. 1C). ACTH incubation did not alter the output of labelled products, while incubation with insulin showed a significant divergence of de novo labelled material in the media already at 6 hrs.

**Figure 2:**
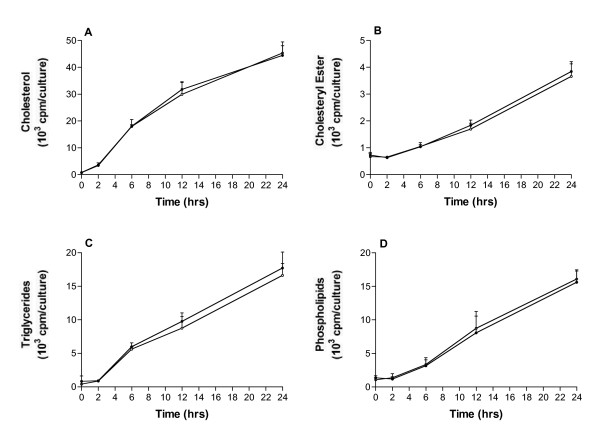
**Contents of labelled lipids over 24 hrs**. HepG2 cells prestimulated with ACTH (100 pM ACTH1-39) were further incubated with ACTH and [^14^C]acetate or [^3^H]glycerol. Labelled cholesterol (A) and cholesteryl ester (B) and triglyceride (C) and phospholipid (D), respectively, were followed during 24 hrs into media. Data represent mean ± SD (N = 6).

When HepG2 cells had continuous access to lipid precursor, de novo synthesised and secreted lipids are accumulated over time in the media (Fig. 2A-D). The presence of ACTH had no effect on secretion of the radiolabelled lipids, cholesterol, cholesteryl ester, triglycerides and phospholipids, at any time-point even though the cells had been prestimulated with ACTH for 24 hrs as well. In separate experiments under identical conditions, handled in parallel with cells for lipid studies, ACTH reduced apoB mRNA as seen previously (data not shown), this ensured us that the HepG2 cells was responsive to ACTH. Similar to the accumulation of de novo synthesised and secreted lipids with continuous labelling were seen with prelabel design and also then there were no effect of ACTH (Data not shown). Cells allowed to run in experimental medium for 48 hrs (prelabel or continuous label) show almost no further accumulation of labelled lipids in media at 48 hrs compared to 24 hrs regardless of presence of ACTH (Data not shown).

**Figure 3:**
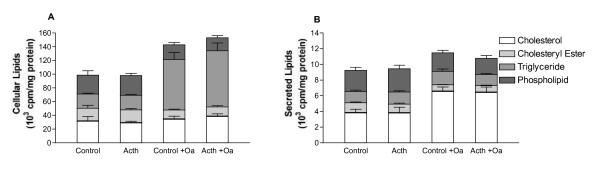
**Contents of labelled lipids in cells and media with HSA or HSA-oleic acid**. HepG2 cells prelabelled with [^14^C]acetate or [^3^H]glycerol was incubated with ACTH (200 pM Synecthen) in 0,4 mM Oleic acid bound to HSA (standard conditions) or in only 0,5% HSA. Labelled cholesterol and cholesteryl ester and triglyceride and phospholipid, respectively, were evaluated after 24 hrs in cells (A) and media (B). Data represent mean ± SD (N = 4-6).

Lipid contents in cells and media have further been studied at 4 and 16 hrs of ACTH stimulation. There was no obvious effect of ACTH on cellular or secreted labelled lipids at these times intervals as well (Data not shown). Oleic acid was used in the experimental medium since it is known to increase stability of apoB and hence increase synthesis of apoB containing lipoproteins [12]. When oleic acid was excluded from the experimental medium there was a change in output of radiolabelled lipids in cells and media, as expected. However, ACTH did not change synthesis or secretion of labelled lipids in the absence of oleic acid either (Fig. 3A and 3B).

## Discussion

In the present study we further confirmed our hypothesis that ACTH lowering apoB containing lipoproteins in humans mainly via the inhibition of hepatic apoB synthesis but not influence cellular lipid synthesis, which is compatible to our recent discovery that lipids was handled just as efficiently regardless of the reduced apoB secretion in response to ACTH administration, suggesting that fewer but more lipid rich lipoproteins are synthesised from the small intestine during ACTH influence [10].

We have previously reported that ACTH strongly reduce apoB synthesis and secretion, with almost no further accumulation at 48 hrs [11]. This was also seen with secreted lipids in this case, especially with prelabel design, suggesting that lipids and lipoproteins may be subject to recirculation [17]. We have not examined the catabolism of nascent lipoproteins, and the possibility of variation in the reuptake of nascent lipids and lipoproteins cannot be ruled out, however we don't think this mechanism can explain the results seen in this study because ACTH do not affect LDL-receptor mediated uptake or mRNA levels of LDL-receptor and scavenger receptor BI receptor [11]. Further, cells almost reach confluence at 48 hrs of lipid accumulating and thus, several aspects of lipid metabolism may have been altered [18,19]. At neither 24 nor 48 hrs did ACTH influenced the cellular lipid pool or lipids secreted in HepG2 cells. Nor was any affect of ACTH on lipid output seen at earlier time points (4 or 16 hrs). Oleic acid was used in the experimental medium due to its ability to stimulate apoB secretion in HepG2 cells by inhibit intracellular apoB degradation [12]. Previously we have showed that oleic acid increased the level of apoB secreted 3-fold in HepG2 cells, however it could not reverse the reduced apoB secretion in response to ACTH [11]. Supplementation of fatty acids stimulates lipid synthesis, in particular triglycerides, though differences in design, such as time and source of fatty acid, yield somewhat different results [20,21]. The results we saw in response to oleic acid in this study resembles that of others with increased intracellular triglycerides and greater secretion of cholesterol to cholesteryl ester among other things [16,22,23]. Whether oleic acid was present or absent in the experimental medium however did not alter the output of labelled lipids in response to ACTH in HepG2 cells in this study.

The fact that secretion of de novo synthesised lipids was not restrained in parallel with reduced apoB output in HepG2 cells in response to ACTH has also bee seen in the human small intestine in vivo [10]. Although these systems of lipoprotein production may be different the secretory pathway for triglyceride rich lipoproteins in liver and intestine share several characteristics [24-26]. Preliminary results in undifferentiated cultured endothelial cells, Caco2, also support that ACTH do not influence lipid synthesis or secretion directly, but needs to be complemented with studies in polarized cells since they display typical endothelial cell characteristics [27,28]. To further study the size and ratio of lipids per apoB of secreted lipoproteins in response to ACTH both in vivo and in vitro is of interest and such studies are initiated.

We know ACTH administration in humans lowers plasma lipids i.e. cholesterol, phospholipids and triglycerides. The hypolipidemic effect includes all apoB-containing lipoproteins, i.e. LDL, VLDL and the atherogenic lipoprotein (a). Generally this can be due to a decreased synthesis or an increased removal of lipoproteins. In HepG2 cells we have demonstrated that ACTH do not influence LDL receptor activity or synthesis, thus do not support an increased elimination of lipoproteins from the liver. Further, we have concluded that catabolism of apoB48 containing chylomicrons was not enhanced by ACTH in a human postprandial study. All this suggests that catabolism of apoB containing lipoproteins is not the primary way for ACTH to lower plasma lipids. The finding that ACTH selectively suppress apoB mRNA levels and secretion in HepG2 cells is of great interest and supported by the findings in this study, of no influence on lipid synthesis or secretion, suggests that ACTH primarily act by reducing apoB output, that is the number of lipoproteins, consequently lowering plasma lipids in humans by secondary mechanisms. Studies to gain better understanding of mechanisms by which ACTH actually lowers plasma lipids may have clinical interest since we, to the best of our knowledge, postulate that ACTH lowers plasma lipids by different mechanisms compared to conventional lipid-lowering agents.

## Competing interests

The authors declare that they have no competing interests.

## Authors' contributions

Conceived and designed the experiments: NX, MBS and PNE. Performed the experiments: MS and NX. Analyzed the data: MS, XN, MBS, and PNE. Wrote the paper: MS, MBS, PNE and NX. All authors read and approved the final manuscript.
